# Measuring Orthographic Knowledge of L2 Chinese Learners in Vietnam Using a Handwriting Task – A Preliminary Report

**DOI:** 10.3389/fpsyg.2022.784019

**Published:** 2022-02-16

**Authors:** Dustin Kai-Yan Lau, Yuan Liang, Hoang-Anh Nguyen

**Affiliations:** ^1^Department of Chinese and Bilingual Studies, The Hong Kong Polytechnic University, Kowloon, Hong Kong SAR, China; ^2^Department of Chinese Language Studies, The Education University of Hong Kong, Tai Po, Hong Kong SAR, China; ^3^Faculty of Chinese Language and Culture, University of Languages and International Studies, VNU, Hanoi, Vietnam

**Keywords:** handwriting, copying, orthographic knowledge, L2 Chinese, Vietnam

## Abstract

In the current study, the orthographic knowledge required for writing Chinese characters was assessed among participants with L1 Vietnamese background who learn Chinese as a foreign language. A total of 42 undergraduates were recruited. They were invited to participate in a delayed Chinese character copying task consisting of 32 characters. Their Chinese character reading abilities were also obtained using a character naming task. All the tests were conducted online during the pandemic in 2021. Results indicated that the participants’ accuracy in the copying task was affected by the familiarity of the characters and the number of strokes of the characters. These effects minimized as reading performance increased. In the inter-stroke interval (ISI) analysis, results indicated a significant boundary effect where ISIs between orthographic units were longer than ISIs within orthographic units, showing the participants’ tendency to chunk Chinese characters into functional units when they write. Only high achievers in the reading task demonstrated the use of both large and small grain-size units in writing (i.e., radical-boundary ISI > logographeme-boundary ISI > non-boundary ISI), while the low achievers only used small grain-size units in their writing. We suggest that the delayed copying task incorporated with handwriting measures is an effective method to assess orthographic knowledge of L2 Chinese learners.

## Introduction

The rapid economic development of China has driven a growth in the learning of Chinese language worldwide. However, due to the unique properties of Chinese, learning to communicate using Chinese as a second language is not an easy task.

For example, Chinese phonologically is a tonal language, where lexical tones associate with syllables contribute to meaning differences. Besides, Chinese is usually described as morphosyllabic in which each basic orthographic unit, or character, is mapped onto one syllable and one morpheme (Chao, 1970; Hoosain, 1992). There are about 1,100 syllables and over 3,000 common characters in modern Chinese. That means on average, each syllable corresponds to more than three different morphemes and characters. For example, the syllable [chang2] corresponds to 长<long>, 尝<taste>, and 常<frequent>. Being able to tell that the common syllable [chang2] in [chang2du4] <length> and [chang2jian4]<common> corresponds to different morphemes is important. Otherwise, it will cause confusion when one tries to parse the meaning of multimorphemic words. One of the useful strategies to differentiate homophonic heteronyms is to refer to their orthographic forms. It is, therefore, commonly believed that the learning of the orthographic forms of Chinese will promote the proficiency of using the language (Zhao, 2009).

Orthographically, each Chinese character is a compilation of strokes organized in a square construction. For example, the character 大 [da4]<big> is constructed by putting the three strokes 一, 丿, and ㇏ in a specific pattern. The number of strokes within a character range from 1 to 24 (Shu et al., 2003). The more strokes in the characters, the higher will be the visual complexity. On the other hand, there exists a major group of characters in Chinese called phonetic compounds, which are composed by putting together semantic radicals that give clues to meanings and phonetic radicals that give clues to sound of the host character. For example, the character 筷 [kuai4]<chopsticks> contains the semantic radical ⺮<bamboo>, which gives a clue to the character’s meaning, and the phonetic radical 快[kuai4]<quick>, which is pronounced identically as the host character. Semantic and phonetic radicals are organized in different configurations. The most common configurations are left-right (e.g., 快) and top-bottom (e.g., 筷).

Apart from phonetic and semantic radicals, there is another group of sub-character orthographic units frequently occurring in Chinese characters documented in the literature. In their study reporting the writing errors produced by a Chinese patient with dysgraphia, Law and Leung (2000) reported the observations of errors involving substitutions of logographemes (i.e., stroke patterns in radicals that are spatially separated, such as “土” and “口” in the radical “吉’’)^1^. Similar errors of logographeme deletions, substitutions, and transpositions during Chinese character copying was also reported in Han et al. (2007). The significant role of logographemes in Chinese character writing was observed not only among neurogenic patients but also neurotypical individuals (e.g., Lui et al., 2010; Lui, 2012; Wong and Lau, 2019). For example, using a delayed copying task, Lui (2012) observed that L1 Chinese primary school children demonstrated better performance on copying stimuli with less number of logographemes, after controlling for number of strokes. The “word length” effect was taken as evidence to suggest that logographemes are used by L1 Chinese learners in their writing process.

When individuals start learning a second language, intensive instructions are usually needed. Later on, they are expected to learn new items without instructions by applying their metalinguistic knowledge regarding the second language. With sufficient knowledge of the Chinese orthography, individuals learning Chinese as second language are expected to be able to learn to read and write new Chinese characters independently.

The knowledge of the Chinese orthographic system required for writing Chinese characters include the configuration of the Chinese characters (e.g., Yeh and Li, 2002), a repertoire of the orthographic units of different grain sizes (e.g., Lau, 2019), and the positional consistency of the components (Taft et al., 1999; Lui et al., 2010). Previous studies have documented the significance of each of these areas of orthographic knowledge in learning to read Chinese (e.g., Loh et al., 2018; Chan et al., 2021; Chang et al., 2021; Loh et al., 2021).

The significance of knowledge of configuration of Chinese characters in learning to read Chinese is that it helps to distinguish visually complex characters (e.g., Yeh and Li, 2002). Evidence of the significance of awareness of character configuration in Chinese character learning among CSL learners were reported in previous studies (e.g., Loh et al., 2018, Loh et al., 2021). For example, Loh et al. (2018) conducted a structure identification task on CSL children attending Grade 5–11 in mainstream education in Hong Kong and observed that the children were able to choose the most matched structures (represented in line-drawing figures) of the target characters in over 60% of the time across all grade levels. Similar results were reported in Loh et al. (2021).

The importance of a repertoire of the orthographic units of different grain sizes in learning to write Chinese concerns its role in allowing the decomposition of characters into components so as to reduce the workload of learners’ working memory in the processing. This allows the individuals to more efficiently and effectively recall and retain the orthographic forms of characters than perceiving the characters as a pile of interwoven strokes (Loh et al., 2021). The repertoire was usually tested by asking L2 learners to decompose Chinese characters into components. For example, Shen and Ke (2007) reported that American CSL college students were able to improve from just above 50% scoring rate to over 70% scoring rate after 1 year of study. In another study, Nguyen et al. (2016) also found that a group of Vietnamese students who had studied Chinese for 3 months were able to use components as processing units when learning unfamiliar Chinese characters.

Finally, the significance of positional consistency of the components in learning Chinese characters has been reported among L1 Chinese (e.g., Tong et al., 2017) and L2 Chinese (e.g., Chang et al., 2021; Loh et al., 2021). By using radicals with high positional consistency to create pseudo-characters and radicals with low positional consistency to create non-characters, Chang et al. (2021) observed that L2 Chinese learners showed tendency to choose the pseudo-characters as “more like real characters” and such tendency was observed among those with higher Chinese reading proficiency but not among those with lower Chinese reading proficiency.

Thus far, previous studies that investigated orthographic knowledge of CSL learners mostly relied on tasks that involved close-end questions, i.e., either binary choice (e.g., Chang et al., 2021) or multiple choice questions were given (e.g., Loh et al., 2018; Chan et al., 2021). One of the potential issues associated with close-end questions concern the possible ceiling effect in the data obtained, particularly if the participants have relatively good orthographic knowledge. In the current study, we explored the possibility of using a handwriting task to measure CSL learners’ orthographic knowledge, which potentially can avoid the ceiling effect issues described.

**FIGURE 1:**
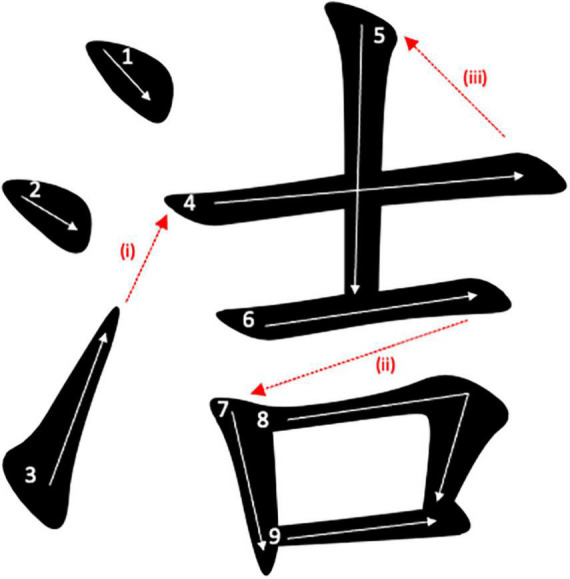
Examples of **(i)** radical-boundary ISI, **(ii)** logographeme-boundary ISI, and **(iii)** non-boundary ISI. The white arrows denote the stroke sequence to write the character.

Using tablets installed with a homebrew Android application that recorded the written responses of the participants using the open-sourced MotionEvent package, Lau (2020b) invited a group of undergraduate students with L1 Chinese background to participate in an immediate character copying task. In the experiment, the inter-stroke intervals (ISIs), measured as the time difference between the end point of a stroke and the start point of the subsequent stroke, in the handwriting production were collected. The results indicated that after controlling for the inter-stroke distance (ISD) (i.e., the linear distance between the end point of a stroke and the start point of the subsequent stroke), the radical-boundary ISIs, i.e., ISIs located at the boundary between the semantic and phonetic radicals, were longer than the logographeme-boundary ISIs, i.e., ISIs located at the boundary between consecutive logographemes, which, in turn, were longer than the non-boundary ISIs, i.e., ISIs located within logographemes. Examples of radical-boundary ISIs, logographeme-boundary ISIs and non-boundary ISIs were given in Figure 1. The significant orthographic boundary effect was taken as evidence to support that radicals and logographemes are functional writing units when people write Chinese characters. Apart from the significant boundary effect observed, it was further found that radical-boundary ISIs of high-frequency characters were shorter than radical-boundary ISIs of low-frequency characters, which is consistent with previous studies that have reported the radical boundary effect in peripheral processing of writing (e.g., Zhang and Feng, 2017).

Similar orthographic boundary effect was observed not only among mature L1 Chinese users but also beginning L1 Chinese learners. Lau (2019) applied a similar immediate character copying task, but with fewer stimuli, on a group of primary school Chinese children. A similar significant orthographic boundary effect (i.e., radical-boundary ISI > logographeme-boundary ISI > non-boundary ISI) was observed, suggesting the unique roles of radicals and logographemes as orthographic units essential for individuals learning to write Chinese character. Finally, it was also reported that children with higher proficiency demonstrated greater flexibility in choosing between radicals and logographemes as the writing units, whereas younger children tend to heavily use logographemes as the writing units. The difference was attributed to the exposures needed for the acquisition of the graphic motor patterns associated with (high-frequency) radicals by concatenating the graphic motor patterns associated with the corresponding constituent logographemes which are unique in Chinese writing (Lau and Yuen, 2019).

Overall, results of previous studies indicated that handwriting measures allowed the observations of orthographic knowledge, particularly the flexibility of using orthographic units of different grain sizes, applied by L1 Chinese users in writing Chinese characters. In the current study, we attempted to examine the orthographic knowledge required for writing Chinese characters among individuals with L1 Vietnamese background who learn Chinese as a foreign language by applying similar handwriting measures. Vietnamese students are unique since they have a demand for understanding Chinese scripts to preserve their culture, which had for a long time previously been documented in Chinese characters. The Vietnamese even invented the *chñ’ Nôm* scripts by modifying Chinese characters to represent native Vietnamese words in the 13th century (Nguyễn Đình Hoà, 1959). In 1910, the French colonial administration required that all public documents be written by the Vietnamese alphabet (Vietnamese: chũ’ Quếc ngñ’; literally meaning “national language script”), a romanization of Vietnamese based on the alphabets of Romance languages. As a result, Chinese characters gradually fell out of use in Vietnam (Zhang, 2009), until recently learning Chinese as a foreign language gradually became more popular because learning the language possibly improve the vocational competitiveness of the learners.

To assess the orthographic knowledge of our participants, they were given a delayed character copying task, which requires better usage of orthographic knowledge to be temporarily stored in the short term memory compared with immediate copying tasks (e.g., Han et al., 2007; Bonin et al., 2015). Given that better orthographic knowledge has been reported to be associated with better reading performances (e.g., Leong et al., 2011; Tong et al., 2017), participants’ performance in a reading test was included in the data analyses. It was expected that individuals with better reading scores, hence better orthographic knowledge, would achieve higher accuracy in the task. Besides, it was expected that similar effect of number of strokes (Lau, 2020b) and character frequency (Lau, 2019, 2020b) observed in L1 Chinese users’ handwriting would also be observed among our participants. Most importantly, it was anticipated that for those who have a better repertoire of the orthographic units of different grain sizes should be better in mastering both radicals and logographemes as writing units. Hence, it was expected that they would demonstrate the significant orthographic boundary effect (i.e., radical-boundary ISI > logographeme-boundary ISI > non-boundary ISI) in their writing.

## Materials and Methods

### Participants

A total of 42 undergraduate students (2 Male and 40 Female, age range from 18 to 22, year of university education range from 1 to 4) majoring in Chinese were recruited in the University of Languages and International Studies, Vietnam National University, Hanoi. Only individuals with no reports of sensory, intellectual and learning problems and no prior training of linguistics and psychology were recruited. All participants reported to have completed at least grade 1–4 of HSK.

### Stimuli and Procedures

Data collection was conducted during the pandemic in 2021. Therefore, each participant was tested individually via a Zoom meeting. The Zoom meeting was video-recorded for later accuracy judgment.

#### Reading Test

A total of 190 characters (40 non-phonetic compounds and 150 phonetic compounds) were selected from the HSK word list. In each trial, a randomly ordered selected character was shown on the screen. The participant was required to read aloud the character. No feedback of accuracy was given. Each correctly named item was given one mark.

#### Delayed Copying Task

A total of 32 Chinese characters, half of them organized in left-right and half of them organized in top-bottom configurations, were selected from the HSK word list. Table 1 shows the demographic information of the stimuli. The participants were invited to use their own smartphones to perform the writing task. A weblink designed for collecting handwriting data using the open-sourced MotionEvent package was given. In each randomly ordered trial, a target character was displayed on the screen for 5 s. Upon the disappearing of the target character, the participant was required to write, using their index fingers^2^, the target character they just saw on his/her smartphone using the browser-based handwriting data collection app. Upon completion of the task, the participant was instructed to submit the handwriting data to the research team via email. Accuracy of each copied item was obtained. Besides, the elapsed time and coordinates each time the fingertip touched or left the device screen were recorded accordingly. The ISIs and the corresponding ISDs were calculated accordingly.

**TABLE 1:** Demographic information of the stimuli of the left-right and top-bottom configurations.

	Left-right configuration	Top-bottom configuration
*N*	16	16
Mean character frequency^#^ (SD)	1.63 (1.36)	1.06 (0.93)
Mean number of strokes (SD)	11.31 (2.89)	11.38 (3.24)

*^#^Character frequency measured as the count of number of words in the HSK level 1–4 vocabulary list containing the target character.*

### Data Analysis

Separate linear mixed effects models with maximal model structure (Barr et al., 2013) were computed using the lme4 package (version 1.1–18.1; Bates et al., 2015) in R (version 3.5.1; R Core Team, 2018) for the accuracy and the ISIs obtained. In the accuracy analysis, character frequency, number of strokes, Configuration, Gender and reading scores were entered as fixed factors to investigate their significance in predicting the copying accuracy. In the ISI model, the ISD, number of strokes, Configuration, Gender, character frequency, radical frequency, logographeme frequency, and regularity of the corresponding ISIs were entered as fixed factors to investigate their significance in predicting the ISIs. By-subject and by-item random intercepts and random slopes were included for each fixed main effect based on recommendations by Barr et al. (2013). Significance was determined using a cut-off point of *t* > 2. The results of the statistical models of accuracy and ISI analyses are summarized in Tables 2, 3 correspondingly.

**TABLE 2:** Results of the model examining the predictors of accuracy of delayed copying.

	Estimate	SE	*t*-value
(Intercept)	1.46	0.15	9.85*
Reading score	–0.003	0.001	−2.59*
Character frequency	0.067	0.029	2.24*
Number of strokes	–0.072	–0.012	−6.24*
Configuration	0.004	0.023	0.34
Gender	–0.008	–0.043	0.29
Reading score: Character frequency	–0.0004	0.0002	−2.03*
Reading score: Number of strokes	0.0003	0.00008	4.86*

*p < 0.05; SE, standard error.

**TABLE 3:** Results of the model examining the predictors of ISI of delayed copying.

	Estimate	SE	*t*-value
(Intercept)	320.37	76.95	4.16*
ISD	0.411	0.049	8.41*
Character frequency	−13.56	2.83	−4.65*
Number of strokes	3.81	1.02	3.76*
Configuration	7.56	6.27	1.21
Gender	6.59	72.93	0.09
Reading Score	0.09	0.56	0.16
BoundaryType (Non vs. Logo)	−31.62	26.83	–1.18
BoundaryType (Logo vs. Radical)	−46.50	37.69	–1.20
Reading Score: BoundaryType (Non vs. Logo)	−0.39	0.19	−2.04*
Reading Score: BoundaryType (Logo vs. Radical)	0.72	0.27	2.67*

*p < 0.05; SE, standard error; Non, non-boundary ISI; Logo, logographeme boundary ISI; Radical, radical boundary ISI.

## Results

### Accuracy of Delayed Copying

The average reading scores obtained was 131.88 with a standard deviation of 29.97. The average accuracy of the delayed copying task was 92.08% with a standard deviation of 7.09. Results of correlation test using Pearson’s *r* indicated strong association between reading scores and accuracy in delayed copying (*r* = 0.55, *p* < 0.01).

Results of LMEM showed that accuracy increased as character frequency increased (0.07 ± 0.03, average count), particularly among those with lower readings scores (interaction of Reading Score/Character Frequency: −0.0004 ± 0.0002). Results also showed that accuracy decreased as number of strokes increased (−0.07 ± 0.01, average count), particularly among those with lower reading scores (interaction of Reading Score/Character Frequency: 0.0004 ± 0.00008). Effect of Configuration and Gender were not significant in predicting Accuracy.

### Inter-Stroke Intervals

Only accurate trials were included in the data analysis. Data from the items with ISIs beyond three standard deviations from the mean (a total of 0.3%) were excluded from the analysis. The results showed that the ISI increased with ISD (0.41 ± 0.05). The longer the ISD, the longer were the ISI. Besides, ISI also increased with stroke number (3.83 ± 1.02), meaning that longer ISIs were associated with characters with more strokes. On the other hand, ISI was observed to decrease with character frequency (−13.02 ± 2.79), meaning that shorter ISIs were associated with characters with higher frequencies.

**FIGURE 2:**
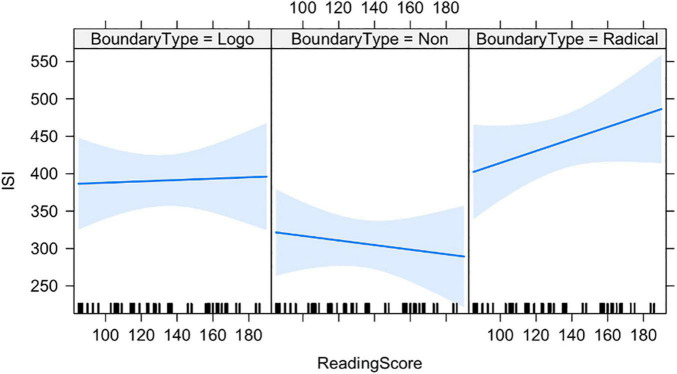
ISI as a function of BoundaryType and Reading Score. Non, non-boundary ISI; Logo, logographeme-boundary ISI; Radical, radical-boundary ISI.

Figure 2 shows the ISI as a function of BoundaryType and Reading Score. The results showed that the ISI increased with Reading Score at the Radical Boundary (interaction of Character Frequency/Between-Radicals: 0.72 ± 0.27) and decreased with Reading Score within logographemes (interaction of Character Frequency/Within-Logographeme: −0.39 ± 0.19). As indicated in the figure, Between-Radicals ISIs increased as Reading Score increased while Within-Logographeme ISIs decreased as Reading Score increased.

Results also showed that ISIs located at the Logographeme Boundaries were significantly longer than ISIs within logographemes (98.22 ± 16.76). Effect of Configuration and Gender were not significant in predicting ISI.

## Discussion

The current study aimed at examining the orthographic knowledge required for writing Chinese characters among individuals with Vietnamese L1 and Chinese L2 by observing their performances in a delayed copying task.

Results showed that participants with lower reading scores were more prone to making errors in the delayed copying task. This strong association between reading and writing performance is consistent with previous studies conducted among L1 Chinese learners (e.g., Tan et al., 2005; Yeung et al., 2011) as well as L2 Chinese learners (e.g., Cao and Perfetti, 2016; Wong, 2018). In general, a strong reading-writing connection was reported among learners of Chinese.

### Accuracy Analysis

In this current study, to understand the association between accuracy of delayed copying and reading score, the processes involved to achieve the requirement of the delayed copying task. Using the logogen model (Ellis and Young, 1996), Bonin et al. (2015) explained the processes involved in delayed copying task and Han et al. (2007) also highlighted the orthographic components involved in Chinese character delayed copying. During each trial of the delayed copying task, a Chinese character was presented. The participants were required to copy the character upon the disappearance of the Chinese character. To achieve this, if the character was a familiar item, it would be visually recognized and the corresponding stored item in the orthographic output lexicon would be activated. Subsequently, the sub-character units, such as phonetic and semantic radicals and logographemes, would be temporarily stored in the orthographic output buffer, waiting for the motor execution of writing. During the handwriting phase, the corresponding graphic motor patterns of the sub-character units would be retrieved for the motor execution of writing. In the case that the character was an unfamiliar item, the visual recognition stage would not be possible. Instead, the character would be directly broken down into its sub-character components and temporarily stored in the orthographic output buffer for the motor execution of the writing. Similarly, in the handwriting phase, the corresponding graphic motor patterns of the sub-character units would be retrieved for the motor execution of writing. To facilitate our discussion, the former, which involves familiar characters, is referred as the lexical approach, while the latter, which involves unfamiliar characters, is referred as the non-lexical approach.

It is important to note that the use of non-lexical approach should be more prone to making errors in the delayed copying task. It is because it involves only short term memory and lacks the support from long term lexical memory. The significant effects of character frequency and number of strokes in predicting the accuracy of the task observed in the current study provided support to this notion. High frequency characters should have higher familiarity than low frequency characters, hence are less prone to errors in the delayed copying. Similarly, the more number of strokes in the characters, the higher demand in the short term memory, particularly when unfamiliar characters were encountered, and, therefore, are more prone to errors in the delayed copying as well.

Finally, the significant interaction effect between reading scores and character frequency as well as the significant interaction effect between reading scores and number of strokes indicated that those with better reading scores were less affected by frequency and number of strokes of the stimuli in the delayed copying task comparatively. Among participants with better reading performance, it is likely that they have better lexical knowledge, which allows better usage of the lexical approach in the delayed coping task, as well as better orthographic knowledge, which allows better usage of the non-lexical approach when unfamiliar items were encountered. On the other hand, among participants with weaker reading performance, the insufficient lexical knowledge means they probably have to heavily rely on the non-lexical approach, which has heavy demand of orthographic knowledge, to achieve the task requirements. Therefore, the lower accuracies achieved indicated that they have insufficient orthographic knowledge too. The insufficient orthographic knowledge may be exhibited in the delayed copying task as (1) having fewer familiar orthographic units with various grain sizes stored, and/or (2) being not flexible enough in using orthographic units with various grain sizes in the processing. The results obtained in the ISI analysis further supported this claim.

### Inter-Stroke Interval Analysis

In the ISI analysis, the results showed that longer ISIs are associated with longer ISDs. This is consistent with previous observations among L1 Chinese users (e.g., Lau, 2019, 2020b). Such observation should not be surprising, since the longer the physical distance that the fingertip travels during the writing process, the longer time should it take to achieve the travel.

Besides, the results showed that longer ISIs are associated with characters with more strokes. Again, this is consistent with previous observations among L1 Chinese users (e.g., Lau, 2020a,b). Lau (2020b) suggested that the positive correlation between number of strokes and ISIs may be due to more processing units being temporarily stored in the orthographic output buffer, which induced heavier processing demand during the writing process. This is particularly applicable when the non-lexical approach was used to achieve the requirement of the delayed copying task in the current study.

Moreover, results also showed that shorter ISIs are associated with characters with higher frequency. Once again, this is consistent with previous observations among L1 Chinese users (Lau, 2019, 2020a). Lau (2020a) suggested that the character frequency effect may reflect that the time needed to retrieve and/or plan for the writing of high frequency writing units are shorter than that of low frequency writing units. Alternatively, it is also possible that the delayed copying of low frequency characters relies heavily on the non-lexical approach, which induces heavier demands in short term memory given the lack of support from long term lexical memory. Hence, the handwriting process would also be affected accordingly, given the nature of cascaded relationship between central processing and peripheral processing of writing (e.g., Qu et al., 2011; Roux et al., 2013).

Finally, the results showed longer ISIs at radical boundaries and logographeme boundaries in general. Such significant boundary effect was reported in previous reports among L1 Chinese users (Lau, 2019, 2020a,b). Lau (2020b) suggested that the longer ISIs located at the orthographic unit boundaries were attributed to longer time required to retrieve the writing units. The significant orthographic unit boundary effects observed in the current study, therefore, indicated that all the participants chunk the target characters into smaller units when they write the characters. However, unlike typical mature L1 Chinese users, the radical boundaries are comparable instead of longer than logographeme boundary (i.e., radical-boundary ISI = logographeme-boundary ISI), except among the better readers. Only the better readers demonstrated the boundary effect that indicated a better mastery of orthographic units of different grain sizes in their writing (i.e., radical-boundary ISI > logographeme-boundary ISI > non-boundary ISI). It is important to note that a better mastery of orthographic units of different grain sizes does not necessarily mean an overall faster writing speed, hence shorter ISIs in general. Instead, the better mastery of orthographic units of different grain sizes should avoid the heavy reliance of orthographic buffer and/or short term visual memory to fulfill the task requirements. Therefore, individuals who can use both big and small units flexibly in the delayed copying task should demonstrate both higher accuracy in the task as well as the specific orthographic boundary pattern (i.e., radical-boundary ISI > logographeme-boundary ISI > non-boundary ISI) in their handwriting performance.

Our results indicated that among those scored low in the reading task, they showed tendency to use smaller units, i.e., logographemes, as the writing units, while among those score high in the reading task, they demonstrated higher tendency to use both large units, i.e., radicals, and small units, i.e., logographemes, as the writing units. In fact, this mastery sequence from small to larger orthographic units is consistent with previous observations among L1 Chinese learners. For example, Lau (2019) tested a group of grade 1 and a group of grade 5 L1 Chinese children using an immediate copying task. It was observed that only the grade 5 children showed tendency to use both logographemes and radicals as writing units, while the grade 1 children showed tendency to use only logographemes as writing units. It was suggested that the early L1 Chinese learners tend to use smaller writing units, because the smaller units consist of fewer strokes, such that the associated graphic motor patterns are less complex. Besides, it was also suggested that the tendency is related to the fact that there are more units to be learnt when the orthographic units are bigger (e.g., Ziegler and Goswami, 2005; Lui, 2012). Similar explanations should also be applicable to beginning L2 Chinese learners.

When the L2 Chinese learners achieve more advanced levels, they demonstrated higher tendency to use bigger processing units in writing. There are at least three benefits of using radicals as writing units. First, fewer units will be needed to be temporarily stored in the orthographic output buffer, which makes it less error prone in the writing task, independent of the usage of the lexical or non-lexical approach to achieve the requirements of the delayed copying task. It is because the orthographic output buffer is in the common pathway shared by the two approaches. Second, the use of bigger units, i.e., radicals, potentially allows the usage of the phonological information associated with phonetic radicals (e.g., Lau and Ma, 2018; Lau, 2020a) and semantic information associated with semantic radicals (e.g., Law et al., 2005) in supporting the writing process. Finally, the use of bigger units appears to have the advantage of achieving more efficient writing, as indicated in the significant decrease in the non-boundary ISIs observed among those who performed better in the reading task. This may be a result of reducing demands in the orthographic output buffer and/or having support from the phonological and semantic information associated with the radicals. Future studies will be needed to warrant this claim.

### Orthographic Knowledge of Chinese L2 Learners Reflected in the Handwriting Task

In the current study, we attempted to use a handwriting task to measure orthographic knowledge of Chinese L2 learners. In the following, we discussed how the knowledge of the Chinese orthographic system required for writing Chinese characters, including the configuration of the Chinese characters, a repertoire of the orthographic units of different grain sizes, and the positional consistency of the components, can be observed in the handwriting task.

In the delayed copying task conducted, characters of either left-right or top-bottom constructions were selected as stimuli to observe the participants’ awareness of left-right and top-bottom configurations of Chinese characters. Given that the results of both accuracy analysis and ISI analysis indicated no significant effect of configuration in predicting the participants’ performance, it is suggested that the majority of our participants have mastered the awareness of the configurations of Chinese character. Results of error analysis further supported this notion.

**FIGURE 3:**
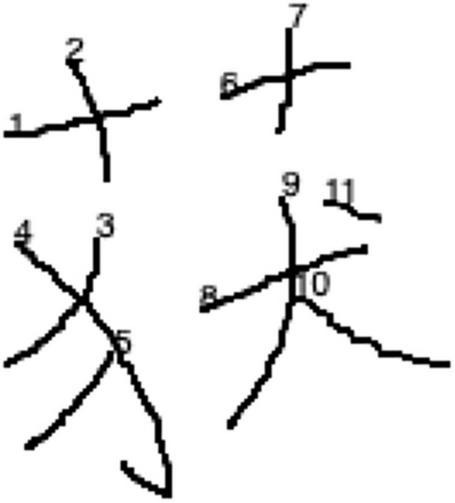
An example of error indicating confusion of character configuration. The order of stroke sequence was indicated next to the onset position of each stroke.

One possible way to detect the confusion of configurations of characters concerns the observations of stroke sequence used by the participants in copying the target characters. One such example was given in Figure 3. As observed in the sequence of writing, indicated by the number next to the onset position of each stroke written, the top-bottom configured character “获”[huo4]<obtain> was written as left-right configured. Nevertheless, such kind of errors was observed to be really exceptional in the current study. Among all the errors produced by the participants, only one involves such confusion of the configuration of the target character. The error was produced by a participant whose reading score ranked second lowest among all participants. Hence, it is suggested that the awareness of common configurations of characters should be relatively easy that even beginning L2 Chinese learners who have completed grade 1–4 of HSK are able to master. It is further suggested that future studies should include other less common configurations of characters, such as enclosed and semi-enclosed configurations (Yeh and Li, 2002; Chang et al., 2021), to obtain a more comprehensive picture of how L2 Chinese learners master the awareness of different character configurations.

Regarding the repertoire of orthographic units of different grain sizes, as indicated in the ISI analysis, the low achievers in the reading test demonstrated the abilities to use logographemes but not radicals in their writing. On the contrary, those with higher reading scores managed to use both logographemes and radicals in their writing. It is suggested that the better readers possess larger repertoires of orthographic units of different grain sizes, which allowed them to perform better in the delayed copying task. Nevertheless, it is noteworthy that the majority (72.3%) of the errors observed in the task involve substitutions or omission of logographemes, which were observed among both weak and good readers. This suggested that despite having relatively larger repertoire of orthographic units, the better readers in the current study still have not master all the orthographic units required for the delayed copying task. This is, in fact, within expectation, as building the repertoire of orthographic units probably requires sufficient exposures and practice.

The third area of orthographic knowledge concerns the positional consistency of orthographic units. Although there was no observation of errors produced by the participants that involved substitutions using logographemes in illegal positions, this may not be sufficient to argue that the participants demonstrated the awareness of positional consistency of orthographic units. Instead, given that the positional consistency of orthographic units should be item-specific, hence the corresponding knowledge should also be exposure-dependent, it is expected that even the better readers in the current study may not have fully mastered the awareness. It is suggested that future studies that include stimuli containing orthographic units of varying degree of positional consistency will be needed to further the investigations.

### Limitations and Future Studies

One obvious limitation of the current study concerns the gender-imbalance of the participants. While this may have potentially created a gender biased issue regarding the results obtained, the female-dominance in the participants actually reflected the common gender-imbalance of L2 Chinese learners in Vietnam. Despite the fact that the insignificant effect of gender observed in both the accuracy and ISI analyses in the current study appeared to support that gender is not a critical factor that affects orthographic knowledge, it is suggested that future studies should try to include more male participants to avoid similar gender-imbalance issue.

Another potential source of limitations concerns the remoteness of the data collection which was conducted through online Zoom sessions. Despite generally good audio and video quality of all the recordings was revealed, it is unclear whether the precision across different smart devices used by the participants may vary, hence potentially affecting the data obtained. The fact that recent studies (e.g., Anwyl-Irvine et al., 2021; Bignardi et al., 2021) reported high accuracy, reliability and validity of data obtained from browser-based and tablet-based experiments, which are independent from the operating systems of the smart devices used, may provide some support to the use of remote data collection via smart devices. It is suggested that a more ideal way is to at least obtain information of smart devices used by the participants and include that as a control variable in the statistical analysis in the future. This is considered particularly important, as remote data collection is getting more popular these days, especially under the city-wide lockdown policies announced by many governments due to the COVID-19 pandemic. The use of browser-based experiments, such as the delayed copying task used in the current study, may help to reduce the difficulties of collecting data face-to-face. In fact, we have further identified other advantages of using browser-based experiments. For example, it allows efficient data collection, which is welcomed by most participants, particularly during the pandemic, as they do not have to physically attend the experiment sessions. It is further suggested that future studies should explore the use of automatized browser-based copying experiments, while at the same time statistically controlled for the potential variability due to precisions of different smart devices, to achieve mass data collection through crowd-sourcing (e.g., Huang et al., 2016). Data obtained from such big-data experiments should yield invaluable results.

Finally, given the success of the current study in using the delayed copying task to measure orthographic awareness of L2 Chinese learners, it is suggested that future studies should make better use of similar handwriting experiments to investigate other areas of orthographic awareness, required for writing Chinese characters, such as positional consistency of orthographic units. Besides, previous experiments also reported significant phonetic regularity effect among L1 Chinese users’ handwriting performance (e.g., Lau, 2020a). Future studies involving experiments of similar construct are recommended to investigate L2 Chinese learners’ knowledge of the functions of phonetic and semantic radicals of Chinese characters. Such experiments should allow the investigation of how L2 Chinese learners master not only the orthographic system but also its interactions with the phonological and semantic systems of Chinese.

## Conclusion

The current study attempted to use a delayed-copying task to measure the orthographic awareness required for writing Chinese characters among L2 Chinese learners in Vietnam. The results of accuracy analysis and ISI analysis indicated that the awareness of character configurations is mastered by most L2 Chinese learners who have completed at least grade 1–4 of HSK. Besides, the results of the ISI analysis further indicated that the weaker readers tend to use only small grain size orthographic units while the better readers tend to use both small and big grain size orthographic units in the delayed copying task. We propose that the use of stimuli with different orthographic properties together with handwriting measures is effective in measuring the orthographic awareness of L2 Chinese learners.

## Data Availability Statement

The original contributions presented in the study are included in the article/Supplementary Material, further inquiries can be directed to the corresponding author/s.

## Ethics Statement

The studies involving human participants were reviewed and approved by the Human Subjects Ethics Sub-Committee of The Hong Kong Polytechnic University (HSEARS20210529002). The patients/participants provided their written informed consent to participate in this study.

## Author Contributions

DL and YL contributed to the conception and design of the study. YL and H-AN organized the data collection and data analyses. DL performed the statistical analysis and wrote the first draft of the manuscript. All authors contributed to manuscript revision, read, and approved the submitted version.

## Conflict of Interest

The authors declare that the research was conducted in the absence of any commercial or financial relationships that could be construed as a potential conflict of interest.

## Publisher’s Note

All claims expressed in this article are solely those of the authors and do not necessarily represent those of their affiliated organizations, or those of the publisher, the editors and the reviewers. Any product that may be evaluated in this article, or claim that may be made by its manufacturer, is not guaranteed or endorsed by the publisher.

## Footnotes

1 Distinguishing between logographemes and radicals is sometimes difficult, as some logographemes share the same orthographic forms with radicals. For example, the logographeme 忄 in the character 筷 shares the same orthographic form with the semantic radical 忄<mental−related> in the character 憶 [yi4]<recall>. In the latter case, the radical 忄 gives clues to the meaning of the target character 憶, whereas in the former case, the logographeme 忄 contributes neither to the sound nor the meaning of the target character 筷. In this study, logographemes and radicals were defined according to whether they carry functions of semantic and phonological in the character contexts or not.

2 It is noteworthy that finger-writing differs from stylus-writing according to the literature (Prattichizzo et al., 2015). The difference, however, has been reported to be related to the precision of strokes in the writing instead of the overall outcome, hence the accuracy, of the writing. In other words, finger-writing mainly affects the degree to which precision of motor execution of writing can be achieved. In the delayed copying task used in the current study, the required orthographic knowledge in the processing, including the configuration of characters, the repertoire of orthographic units with different grain sizes, and the positional consistency of orthographic units, should be common no matter finger-writing or stylus-writing is used.
